# Applications of Platelet Concentrates (PCs) in Regenerative Onco-Urology: A Systematic Review of Literature

**DOI:** 10.3390/ijms251910683

**Published:** 2024-10-04

**Authors:** Andrea Gottardo, Gabriele Tulone, Nicola Pavan, Fabio Fulfaro, Valerio Gristina, Tancredi Didier Bazan Russo, Ornella Prestifilippo, Francesco Claps, Lorena Incorvaia, Antonio Galvano, Antonio Russo, Alchiede Simonato

**Affiliations:** 1 Department of Precision Medicine in Medical, Surgical and Critical Care, University of Palermo, 90127 Palermo, Italy; andrea.gottardo@unipa.it (A.G.); gabriele.tulone@gmail.com (G.T.); fulfaronc@hotmail.com (F.F.); valerio.gristina@unipa.it (V.G.); tancredididier.bazanrusso@unipa.it (T.D.B.R.); ornella.prestifilippo6@gmail.com (O.P.); lorena.incorvaia@unipa.it (L.I.); antonio.galvano@unipa.it (A.G.); antonio.russo@usa.net (A.R.); alchiede.simonato@unipa.it (A.S.); 2 Urology Clinic, Department of Medical, Surgical and Health Sciences, University of Trieste, 34127 Trieste, Italy; claps.francesco@gmail.com

**Keywords:** radiation cystitis, hemorrhagic cystitis, actinic cystitis, urethral obstruction, urethral stenosis, platelets concentrates, platelet-rich plasma, PRP, onco-urology, systematic review

## Abstract

*Objective*: To assess the effectiveness of Platelet Concentrates (PCs) in the contest of Hemorrhagic, Actinic, and Radiation Cystitis, plus Urethral Obstruction or Stenosis. *Eligibility criteria*: Open article in English or Italian regarding in situ applications of PCs for the selected pathologies. *Information sources*: MEDLINE, Cochrane Library, and ELSEVIER. *Risk of bias*: High (and discussed). *Methods for synthesis of results*: Selection of relevant contents, resumed by digital tools, checked by authors and used throughout the manuscript. *Included studies*: 13 screened articles + 7 personal sources + 37 “extra” articles. *Synthesis of results*: Pre-clinical and clinical studies demonstrated substantial symptom relief, mucosal restoration, and improved growth factor levels, reducing recurrence rates and complications. However, preparation protocols and results varied among studies. *Limitations of evidence*: Frequent low-quality studies with mall sample size, plus heterogeneous experimental setups and nomenclature/preparations. *Interpretation*: PCs demonstrate promise due to their bioactive components, enhancing tissue repair and reducing inflammation with no significant adverse events. Despite positive outcomes in pre-clinical and clinical studies, variability in preparation protocols and small sample sizes, together with inconsistent results, highlight the need for high-quality research to validate PCs’ clinical efficacy and cost-effectiveness.

## 1. Introduction

Platelet Concentrates (PCs), of which Platelet-Rich Plasma (PRP) is the best known in the literature, are both autologous and heterologous/allogenic formulations that have shown significant promise as a novel therapeutic approach for various conditions, even within the urological field [1,2,3,4,5]. PCs contain an array of bioactive molecules—i.e., cytokines, chemokines, and most of all growth factors (GFs) (including but not limited to platelet-derived growth factors (PDGFs), transforming growth factor-β (TGF-β), vascular endothelial growth factor (VEGF), epidermal growth factor (EGF), and insulin-like growth factor-1 (IGF-1)) [6]—which play crucial roles in tissue repair [7]. In particular, platelet GFs work by promoting and guiding tissue regeneration; thus, they facilitate healing processes in damaged tissues [8].

PRP is known not only for its regenerative properties but also for its significant anti-inflammatory effects [1]. These properties are particularly beneficial in treating various urological conditions, as well as other medical conditions where inflammation plays a critical role. Indeed, by decreasing inflammation, PRP can reduce symptoms such as swelling, redness, and heat, which are commonly associated with inflammatory responses, thus improving patient quality of life (QoL) [2].

Clinically, PRP is used to enhance the healing process [9] since the bioactive molecules released by platelets’ granules can promote angiogenesis and increase blood flow and wound oxygenation [10,11]. PRP seems to also contain mesenchymal stem cells (MSC), which contribute to the wound-healing process [7,12,13]. Moreover, macrophages and neutrophils recruited by platelets play an important role in starting an initial pro-inflammatory response, followed by a late anti-inflammatory phenotype that leads to the release of anti-inflammatory factors [14]. Finally, PRP can eliminate neuropathic pain, primarily due to factors released from platelets and stem cells that initiate the complex cascade of wound healing events [15].

In onco-urology, the application of PRP is generally performed through direct instillation into the bladder, making it a minimally invasive procedure. This method has been well-tolerated by both rat models and patients, showing positive results in reducing symptoms and improving bladder function in several conditions [1,7,16]. Talking about onco-urological conditions that may be treated with PRP applications, three of the most common but challenging pathologies seen in our departments are hemorrhagic/actinic cystitis, radiation cystitis, and urethral obstruction/stenosis—i.e., all adverse events (AEs) that might be due to neoplastic pathologies or may arise after oncological treatment.

In detail, hemorrhagic cystitis (HC) is a sterile cystitis marked by persistent bleeding from the bladder mucosa, leading to macroscopic hematuria [17]. It commonly occurs after allogeneic hematopoietic stem cell transplant (allo-HSCT) in patients with hematological malignancies due to BK virus (BKV) infections [18,19], or even in patients treated with oxazaphosphorine chemotherapeutics (i.e., ifosfamide or cyclophosphamide (CYP)) or pelvic radiotherapy (RT) [2,20]. The second condition, known as actinic cystitis (AC), is more prevalent and accounts for approximately 95% of idiopathic hematuria cases; it is induced by a chronic inflammatory process that affects the bladder mucosa and extends to the submucosal vascular network, leading to its characteristic symptoms [20]. However, about 50% of patients undergoing allo-HSCT develop BKV viruria (notably children [21]); of these, 5–40% will develop active HC, ranging from microscopic hematuria to severe bladder hemorrhage with renal impairment. Speaking generally about HC, it constitutes 27% of urological consultations and 4–20% of hospital consultations for hematuria. Radiation cystitis accounts for 5–10% of these cases. Approximately 50% of patients with RT-related HC for prostate cancer require invasive procedures. Up to 40% of patients on oxazaphosphorine chemotherapy develop HC, though some preventive measures (such as hyperhydration, bladder irrigation, sodium hyaluronate, and cranberry products) seem to reduce this AE [17].

Urethral stricture or stenosis (US) is another common complication of RT for both men and women patients [17,22]. Particularly, Dirk et al. [18] conducted a health-related QoL assessment in male patients with urethral stenosis due to prostate RT, discovering that, even if treated with urethroplasty, endoscopic treatment, or indwelling catheter, still two third of the interviewed patients reported frequent or total incontinence and required daily pad use, while between 85% and 90% of patients reported various sexual dysfunction. Finally, depressive symptoms and fatigue were reported by 41.4% and 60.9% of patients, respectively.

As a result of some preliminary evaluations, we found indeed that all these urological AEs of cancer treatments could be addressed with the use of PCs. Therefore, the purpose of this Systematic Review is to comprehensively evaluate what the scientific literature says about the application of such compounds for the treatment of these diseases in the hopes of obtaining sufficient results to justify their subsequent use in a dedicated clinical study. To do so and to overcome the preliminarily found paucity of articles specifically covering this topic, we systematically collected all the available articles regarding the objects of our research, independently by their nature—indeed, we included commentaries, reviews, pre-clinical and prospective studies to be able to have the most comprehensive view possible about the available knowledge at our disposal through the major scientific databases.

## 2. Methods

### 2.1. Registration and PRISMA Documents

This Systematic Review is registered with the following PROSPERO ID: CRD42024576751. Moreover, this study follow the Preferred Reporting Items for Systematic reviews and Meta-Analyses (PRISMA) guidelines (https://www.prisma-statement.org/, accessed on 1 May 2024); in particular, the Flow Diagram is shown in Section 3, while the Checklist is reported in Supplementary Figures S1 and S2.

### 2.2. Search Strings

The following sources were screened, using the reported search strings and downloading all the resulting papers to be further screened. No additional filters were used.

- MEDLINE via PubMed (https://pubmed.ncbi.nlm.nih.gov/, accessed on 24 April 2024)
  ◦ (“Radiation Cystitis” OR “Hemorrhagic Cystitis” OR “Actinic Cystitis” OR “Urethral Obstruction” OR “Urethral Stenosis”) AND (“Platelet-Rich Plasma” OR “PRP” OR “Platelet-Rich Fibrin” OR “PRF” OR “Platelet Concentrate” OR “Platelet Gel” OR “Platelet Derivatives”)
- CochraneLibrary (https://www.cochranelibrary.com/, accessed on 24 April 2024)
  ◦ (see Table 1)

**Table 1:** Cochrane Library search string.

ID	Search	Hits
1	Radiation Cystitis	188
2	Hemorrhagic Cystitis	164
3	Actinic Cystitis	2
4	Urethral Obstruction	358
5	Urethral Stenosis	444
6	Platelet-Rich Plasma	3418
7	PRP	3658
8	Platelet-Rich Fibrin	1441
9	PRF	1685
10	Platelet Concentrate	848
11	Platelet Gel	496
12	Platelet Derivatives	2928
13	#1 OR #2 OR #3 OR #4 OR #5	1065
14	#6 OR #7 OR #8 OR #9 OR #10 OR #11 OR #12	10,003
15	#13 AND #14	7

- ELSEVIER via ScienceDirect (https://www.sciencedirect.com/, accessed on 24 April 2024, three different searches for the limit set up to eight Boolean)
  ◦ “Radiation Cystitis” AND (“Platelet-Rich Plasma” OR “PRP” OR “Platelet-Rich Fibrin” OR “PRF” OR “Platelet Concentrate” OR “Platelet Gel” OR “Platelet Derivatives”);
  ◦ (“Hemorrhagic Cystitis” OR “Actinic Cystitis”) AND (“Platelet-Rich Plasma” OR “PRP” OR “Platelet-Rich Fibrin” OR “PRF” OR “Platelet Concentrate” OR “Platelet Gel” OR “Platelet Derivatives”);
  ◦ (“Urethral Obstruction” OR “Urethral Stenosis”) AND (“Platelet-Rich Plasma” OR “PRP” OR “Platelet-Rich Fibrin” OR “PRF” OR “Platelet Concentrate” OR “Platelet Gel” OR “Platelet Derivatives”).

### 2.3. Eligibility Criteria and Screening Results

To be eligible, articles must be (1) regarding selected pathologies, (2) regarding in situ applications of PCs, (3) open full text, and (4) written in English or Italian. Articles that do not have all these characteristics were excluded from our results. Article selection was manually and independently made by two authors (Andrea Gottardo + Gabriele Tulone), and their work was further assessed by a third author (Antonio Galvano). All the screening phase is summarized in Section 3; as might be seen there, we included 13 screened studies, 7 personal sources, and 37 “extra articles”, i.e., the most relevant bibliographic citations of the included plus results from other ad hoc research. The 13 screened studies, i.e., the core of this systematic review, are listed in Section 3 too.

### 2.4. Data Extraction and Interpretation

*Criteria* → To find and collect any part of the retrieved articles about PCs and the three pathologies under study, i.e., Radiation Cystitis + Hemorrhagic (Actinic) Cystitis + Urethral Obstruction (Stenosis).

*Round I* → Data were manually and independently extracted from the results of the systematic literature screening by two authors (Andrea Gottardo + Gabriele Tulone), and their work was further assessed by a third author (Antonio Galvano). Once the raw data were collected from each article, ChatGPT was utilized to make Italian draft summaries, or DeepL was employed for translation if the texts were sufficiently concise. The output of both tools underwent review by the authors to produce final summaries in Italian, used during the writing phase.

*Round II* → Data were extracted from other articles already in possession of the authors or from the most relevant bibliographic citations of the included studies. All the papers underwent the same process as seen in the previous step.

*From data to information* → Italian summaries or translations were organized and analyzed and then distributed into sections of this systematic review based on their topic or content (e.g., basic information about the pathologies in Section 1, results obtained via pre-clinical or clinical studies in Section 3, insightful considerations about PCs applications in Section 4, and so on). The authors further interpreted all the collected data and integrated the resulting information into the paper’s text. All the information thus collected is correctly highlighted by the relative reference to underline the difference between the gathered information and our considerations or comments.

### 2.5. Criteria for QA

Articles were manually and independently assessed by two authors (Andrea Gottardo + Gabriele Tulone), and their work was further evaluated by a third author (Antonio Galvano). Once understood the type of study [23,24], we differentially assessed the results regarding solely animal subjects (i.e., pre-clinical studies) and the ones regarding solely human or both human and animal subjects. In the former case, we opt for the Systematic Review Centre for Laboratory Animal Experimentation (SYRCLE) risk of bias tool [25], giving 0 points for “no”, 1 point for “unclear”, and 2 points for “yes”; thus, this quality scale goes from a minimum of 0 to a maximum of 20 points. In the latter case, to first assess the grade of recommendation and level of evidence by the type of study, we adopt the Modified presentation of the Oxford Centre for Evidence-Based Medicine (OCEBM) levels of evidence scale [26]. Then, to properly evaluate the case series articles, we opt for the Quality Assessment Tool for Case Series Studies made by the National Institutes of Health—National Heart, Lung, and Blood Institute (NIH-NHLBI) [27], giving 0 points for “no” and 1 point for “yes”; thus, this quality scale goes from a minimum of 0 to a maximum of 9 points.

## 3. Results

### 3.1. PRISMA 2020 Flow Diagram, Table of Results and Quality Assessment (QA)

**Figure 1:**
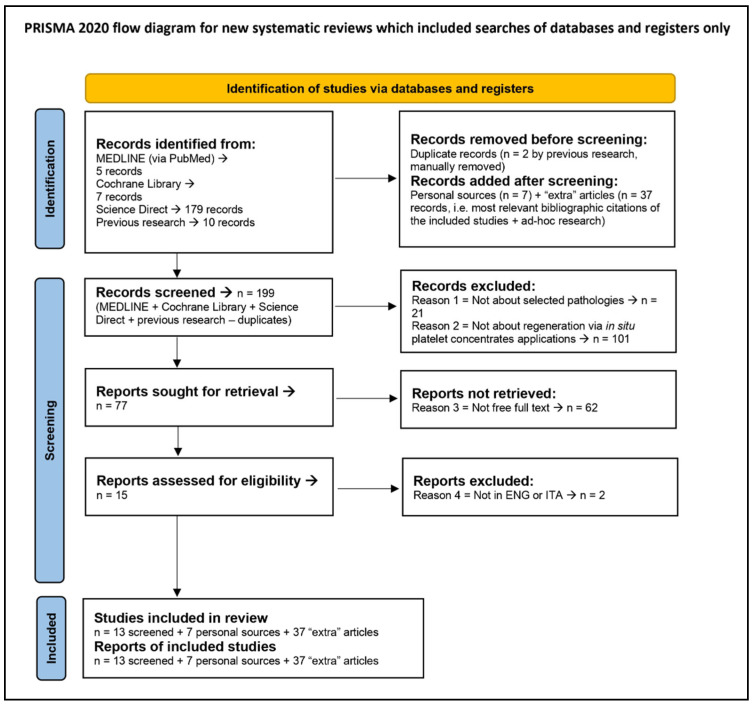
PRISMA 2020 flow diagram.

The official diagram is shown in Figure 1, while the results of the systematic review of the literature and the relative QA results are presented in Table 2 (see “Section 2.5. Criteria for QA” for insight into the scales therein used). To note, four studies [7,12,16,28] discussed a more commonly evaluated pathology in the field of PCs’ applications in urology, i.e., interstitial cystitis (IC). Although we usually excluded studies regarding IC in the study selection phase, these selected four papers are still included due to the contemporary analysis of the pathologies of our interest. Notably, Jhang et al. [7] study was evaluated as low-grade and low-level, though it is actually a clinical study because instead of using information regarding IC treatment, we used information about the scientific field; thus, we treated and consequently evaluated it as a review. To highlight this issue, in Table 2, we marked this study with two asterisks (*). Moreover, the review by Trama et al. [28] reported the insightful pre-clinical study by Chen et al. [29]. Therefore, since we discuss this latter study in Section 3.2, and unlike all the other referenced articles not listed here, we added this pre-clinical study to the table of results, which now reports 14 and not 13 articles (as stated in Figure 1) due to this reason.

### 3.2. Hemorrhagic, Actinic and Radiation Cystitis Treatment with PCs

As said, HC is a significant pathology that may have different etiologies but that occurs in up to 5% of patients following pelvic RT [34]. Although advancements in radiation techniques, such as intensity-modulated radiation therapy (IMRT), may reduce bladder toxicity, comprehensive long-term data are still lacking [35,36]. Radiation-induced endothelial cell damage and perivascular fibrosis lead to tissue ischemia and vascular obliterative endarteritis, resulting in a high risk of hematuria or lower urinary tract symptoms (LUTS) like urinary frequency, urgency, and pelvic pain [37]. Usually [34], the clinical management of these three kinds of cystitis consists of discontinuation of anticoagulant if on therapy, evacuation of bladder clots, continuous bladder irrigation, and blood transfusion as required. However, sometimes this is not sufficient. In this case, invasive surgery techniques, such as urinary diversion or radical cystectomy, are necessary, but they often bring a worsening of the QoL and a high mortality rate [38]. In his clinical study (shown in the Results section of this systematic review), Masieri [30] indicated alternative, less invasive management options, as intravenous cidofovir, fluoroquinolone antibiotics, hyperbaric oxygen therapy, and intravesical therapies, but these strategies have shown several limitations. In truth, even fibrin glue was mentioned, showing good clinical results [39], but the relatively low quality of the study and the difficulties to obtain this blood derivative let the author to opt for PRP for his clinical trial, since it was already known in the literature for its multiple applications as a stimulant of the healing process [40,41,42,43,44] and whose bladder instillation has already been successfully tested both in animal and human patients [7,16].

Similar results were seen comparing intravesical injections of PRP and botulinum toxin A (BoNT-A): as also reported by Kuo et al. [12], the application of BoNT-A is a recognized therapy in several areas of urology; however, this has not been shown to be particularly effective either in in vitro studies [45] evaluating it in single, or in in vivo studies [46] comparing it with PRP, where the latter demonstrated significantly better results and a complete absence of AEs instead.

Indeed, several studies have investigated the use of PRP as a novel, less invasive, and effective treatment for these three kinds of cystitis. The rationale behind this approach relies on the release of platelet content, which, as said, can help restore the bladder mucosa and submucosal vascular network, thereby alleviating symptoms and reducing hematuria. This happens because platelets, once activated through binding of several agonists to platelet G-protein-coupled receptors (GPCRs) or immunoreceptor tyrosine-based activation motif (ITAM) complexes, can release their granules in a controlled way, thus being able to coordinate all the healing process [47]. Additionally, the affordability, ease of obtaining, and easy delivery of PCs through direct instillation to the bladder wall pushed researchers to further test these compounds for the treatment of such lesions [7].

Delving into details of cystitis treatment, the results of our endeavor show two preclinical studies [16,32], two clinical studies [20,30], and a review [28]. Starting with the pre-clinical studies, the articles of both Dönmez [16] (14 points out of 20 in our QA scale) and Ozyuvali [32] (11 points out of 20) assessed the effects of PRP in rabbits and rats, respectively, in which cystitis was induced by intravesical instillations of CYP. For Dönmez, the results show that PRP instillation significantly reduced macroscopic hemorrhage and hematuria in the CYP-treated groups while increasing leukocyte infiltration and edema; to be fair, this reduction was also observed in the hydrochloric acid (HCl)-treated group (tested to simulate IC), but statistical significance was not achieved here. In any case, histological analysis showed that PRP treatment significantly increased the mitotic index in the urothelium and the proliferative response of Proliferating Cell Nuclear Antigen (PCNA)-positive cells, indicating an effect on tissue regeneration. The same result is not seen by Ozyuvali, but it is our opinion that this depends by the experimental timeline: indeed, Dönmez analyzed the treated bladders 48h after the PRP instillation, while Ozyuvali only 6h after the treatment; thus, it is probable that the bladders did not have enough time to regenerate their walls, even if stimulated by PRP.

Abbruzzese [20] (Grade C, level 4—6 points out of 9) conducted a clinical study on nine patients with actinic cystitis treated by bladder instillations with allogenic platelet lysate (PL) for 3 months. The methodology involved intensive treatment in the first month, followed by a progressive reduction in the frequency of installations. The results showed a gradual and constant improvement in patient symptoms over the course of treatment. After three months, a complete resolution of symptoms and restoration of normal bladder mucosa was observed in 8 out of 9 patients. Only one patient continued to present low-grade symptoms. Biopsies were also performed on the patients, which confirmed the restoration of the normal mucosa and the reduction of the inflammatory process. According to Abbruzzese, treatment with PL is undemanding for patients and free of significant AE, observing a substantial improvement in the symptoms of hemorrhagic actinic cystitis.

Masieri’s clinical study [30] (Grade C, level 4—4 points out of 9) also used intravesical administration of PRP (autologous, in this case) to treat 10 consecutive cases of BKV-induced HC. And again, PRP led to a significant reduction in urinary symptoms and pain, with a rapid suspension of post-operative analgesic treatments. Specifically, Masieri obtained a 60% complete response (CR), plus a 30% partial response (PR) and 10% no response (NR) in just one month after therapy. Therefore, even with the limitation of the limited number of patients and the short follow-up, this study shows promising results for the safety and efficacy of the PRP treatment for HC.

Finally, Trama’s review [28] explored the application of PRP for the treatment of Bladder Pain Syndrome (BPS)/IC. Again, although IC is not among the diseases analyzed in this systematic review, nevertheless, the pre-clinical article therein analyzed reports the same setup yet seen by our result. Indeed, he showed the yet-seen Dönmez study [16], plus the study by Chen et al. [29] (11 points out of 20), where the mucosa and submucosa thickness, the proliferation of normal human fibroblast cells (HFCs), the intervals between micturition, plus the expression of cell junction-associated protein zonula occludens 2 (ZO-2) and of the interleukin-6 (IL-6) was assessed to evaluate the efficacy of intravesical instillation with PRP and/or hyaluronic acid (HA) in mouse models of CYP-induced cystitis. The result shows that the PRP group ameliorates all the evaluated markers, even outperforming HA-treated groups; moreover, when PRP was combined with HA, the result was always worse than the ones obtained with solely PRP. Finally (and interestingly), when they first analyzed healthy human skin fibroblast cells (HFCs) and not the biopsies of damaged bladders, they found that PRP stimulated in vitro proliferation in a dose-dependent manner (1–5%) but that a higher concentration (10%) can slow down the physiological proliferation rate of these healthy cells. It is our opinion that this aspect, even if only found in this study, shall deserve further attention through new, ad hoc in vitro studies.

### 3.3. Urethral Obstruction or Stenosis Treatment with PCs

Post-surgical healing of US is another onco-urological area in which we want to assess the PRP effectiveness. This post-surgical healing phase is crucial in preventing recurrence, which is significantly high in US cases. PCs have emerged as a promising adjunctive therapy to enhance tissue repair and reduce recurrence rates after US surgical treatment [8,31]. In the case of US, PRP (studied in this context for the first time by Gul et al. [48]) could reduce the recurrence rate of stenosis by inhibiting TGF-β1. This factor, present in PRP, promotes the fibrotic process. Using PRP together with a TGF-β1 inhibitor, tissue healing could be promoted without promoting pathological fibrosis, as demonstrated by in vitro studies [49,50]. Pre-clinical and clinical studies have shown that PRP can have a preventive effect on US formation and reduce the recurrence rate of bulbar stenosis, as well as improve the outcomes of mucosal graft application [51,52,53]. Although great progress has been made and several techniques have been developed to improve the repair of urethral lesions, complications related to the operation, such as recurrence of strictures and urethrocutaneous fistulas (UCF), remain frequent. Among minimally invasive treatments, the most popular are local tissue flaps and fibrin glues [54,55,56,57]. In this context, the use of Platelet-Rich Fibrin (PRF) has been proposed as an alternative to prevent UCF after urethral repairs [58]. The PRF, first described by Choukroun et al. [59], is a particular kind of PC: differently from the liquid PRP, it has a stronger, fibrous composition due to the high fibrin component, but it is still a great source of GFs that can promote wound healing and collagen synthesis [6,60].

Delving into details, our systematic review outlined a pre-clinical study made by Soyer et al. [8] (8 points out of 20) where they assessed the effects of PRF on GF levels in urethral repair through 18 Wistar albino rats: these were divided into three groups, control (CG), sham (SG), and PRF (PRFG); the SG group underwent a vertical incision and repair of the penile urethra, the PRFG group had PRF applied to the repair site, and the CG was used to obtain the control sample of the incised urethra. After 24 h, tissue samples were analyzed for GF levels. The results indicated that PRF significantly increased the levels of TGF-β-R and VEGF in the urethral tissue compared to the SG group. No significant differences were found in EGFR levels between the groups. These findings suggest that PRF’s beneficial effects on urethral repair might be mediated through mechanisms involving these key GFs and, therefore, the potential of PRF as a valuable adjunctive therapy in urethral surgeries, promoting efficient and robust healing while minimizing complications like UCF.

### 3.4. PRP Preparation Protocols

Abbruzzese [20], Dönmez [16], Trama-reported [28] study by Chen [29], Masieri [30], and Ozyuvali [32] (but not Soyer [8]) report enough details about the PCs’ preparation protocol used. Table 3 synthetizes what they have performed.

As we can see, among the six pre-clinical and clinical studies reported in this systematic review, five of them reported the protocol used to obtain their PCs. However, although all five of the studies reported in Table 3 treated one of the types of cystitis, we analyzed that all of the protocols differed from each other (with the exception of those used by Dönmez [16] and Chen [29]); therefore, although they all treated one type of cystitis, all of them used a different kind of PCs.

**Table 3:** Retrieved preparation protocols.

Article	PC’s Name	Preparation Protocol
Abbruzzese, et al. [20]	PL	(1) 4.4 mL of CaCl was added to every 100 mL of platelet apheresis, with platelet concentration = 1 × 10^9^ ± 20%/L. (2) The mixture was placed in a thermostatic bath at 37 °C for 60 min under stirring. (3) Once the platelet clot appeared, the content was aliquoted, and pH values were measured (6.4–7.4); then, the aliquots were stored at −40 °C. (4) After 7 days, the confirmation of sterility made the product available for clinical use.
Dönmez, et al. [16]	PRP	Protocol by Nagae et al. [61]: (1) Via the intracardiac waist, a mean of 4.5 mL (3.5–6 mL) of whole blood was withdrawn and injected into 4.5 mL tubes containing 3.2% citrate. (2) Once it arrived in the laboratory, centrifugation was performed at 1500 rpm (250× *g*) for 10 min to separate the discharged supernatant and the platelet-containing buffy coat. (3) The buffy coat was centrifuged at 3000 rpm (1000× *g*) for 10 min to separate the Platelet-Poor Plasma (PPP) and to precipitate platelets. (4) The PPP was discharged, and a mean of 1 mL (0.8–1.2 mL) of PRP was obtained.
Chen, et al. [29]	PRP	Protocol by Nagae et al. [61]:
Masieri, et al. [30]	Platelet gel	(1) First centrifugation of 250 cc of platelet apheresis at 200× *g* for 20 min. (2) Second centrifugation at 2000× *g* for 20 min at 24 °C, thus obtaining 50 mL of sterile PRP. (3) 10 mL of plasma is added, and the product is preserved at 4 °C. (4) About 30 min before use, 10 mL of 10% calcium gluconate was added to activate platelet [NDR: thus, obtaining platelet gel].
Ozyuvali, et al. [32]	PRP	(1) 3 mL of blood was withdrawn and collected into a blood tube containing citrate. (2) Immediately after collection, first centrifugation at 1600 rpm for 5 min. (3) Second centrifugation at 2800 rpm for 8 min, gathering 0.3 mL of PRP. (4) After activation, the PRP is used within 3 to 4 h.

### 3.5. AE Assessment

Another important focus of our research concerns the assessment of any AE that treatment via PCs may induce on the evaluated onco-urological conditions. After careful screening of all 13 articles tracked in this systematic review, none of them (whether reviews, pre-clinical studies, or clinical trials) reported any AE, regardless of treatment outcome. Therefore, as Yáñez-Castillo et al. [2] wisely state in their review, “What seems clear is that this treatment does not have significant adverse effects, which sets a precedent for safely conducting clinical trials with a larger sample”.

## 4. Discussion

The use of PCs, both autologous and allogeneic products easily obtainable through centrifugation of venous blood, has shown significant promise as a surgical adjuvant in regenerative applications across various medical fields, including orthopedics, periodontics, rheumatology, dermatology, and urology. The therapeutic effects of PCs are mediated through their bioactive components, including GFs, leukocytes, and fibrin, in varying percentages, depending on the production protocol and specific product. These components allow PCs to modulate immune response, reduce inflammation, promote angiogenesis, and enhance tissue regeneration. PRP, especially, shows promise in enhancing tissue repair and reducing pathological inflammation, and its liquid formulation (easily delivered during minimally invasive surgeries) is thought to be particularly useful in the onco-urological conditions assessed in this systematic review, such as hemorrhagic, actinic, and radiation cystitis, as well as in urethral strictures/stenosis.

Indeed, in nearly all the reported studies, PRP is suggested as an effective treatment for those conditions, with positive results in both animal and human subjects, with the exception of Ozyuvali’s study [32]. As discussed, rabbits were sacrificed six hours after PRP instillation, unlike in Dönmez’s study [16], where rats treated similarly were sacrificed 48 h later, leading to successful results. It is our belief that the short timeline in Ozyuvali’s study [32] did not allow sufficient time for bladder wall regeneration after PRP treatment.

Even if our hypothesis is incorrect, we did not find any AEs related to PC applications. Additionally, in 19 human patients with cystitis (reported by Abbruzzese [20] and Masieri [30]), 14 achieved CR, 4 PR, and 1 NR. Notably, Abbruzzese’s multiple PL applications over three months resulted in better outcomes (8 CR, 1 PR) than Masieri’s single platelet gel application with a one-month follow-up (6 CR, 3 PR, 1 NR), suggesting that multiple liquid PC applications may be more effective than a single semi-solid application.

However, we must acknowledge limitations that are common across studies on PCs: low quality, small sample sizes, and non-standardized nomenclature or preparation methods. Only 19 human patients were included across the 13 studies reviewed, emphasizing the limited statistical relevance of the current literature yet underlined by our QA. Furthermore, as noted in Section 3.4, there is an urgent need for standardization in nomenclature and preparation techniques, as even the same pathology is treated with four different products in the five studies reviewed here. This lack of standardization hinders clinical applications, despite research progress, and further delays the wider acceptance of PCs in the scientific community.

Another key point is the safety of PRP in cancer patients. In this context, we sustain the hypothesis made more than once by Spartalis et al. [33,62]: Since it is known that GFs contained in platelets’ granules can stimulate tumor growth, and since platelets take part in the tumor microenvironment as Tumor-Educated Platelets (TEPs) [3], we concur that it is unwise to apply PCs in the presence of tumor cells in the wound bed or in metastatic disease. That said, it seems equally reasonable to both Spartalis [33] and us that, with proper application methods and appropriate contexts, PCs may still be valuable in treating AEs of oncological conditions, as the ones evaluated in this systematic review. Indeed, bladder instillations and subsequent remotion of liquid PCs in cases of resolved neoplastic pathologies not involving the treated organ (e.g., cystitis) seem to offer a safe treatment option to be further evaluated by proper clinical studies, supported by the absence of AEs in our literature review.

While cost-effectiveness analyses are limited in onco-urology, PCs, and particularly PRP (with costs around €350 per preparation in Europe), are known for their cost-effectiveness in various medical fields [63,64]. Therefore, depending on the choice of preparation method and the number of applications, PRP might also be cost-effective in onco-urology.

Lastly, in reviewing the strengths and weaknesses of our study, this is the first systematic review to assess multiple pathologies and types of PCs in onco-urology, offering the opportunity to compare different PCs in varied contexts. Moreover, having noted that different results corresponded with different experimental settings shed light on what, in our opinion, is one of the most critical points of the PCs’ literature; indeed, a simple change in the timeline of an interventional study can exert tremendous influence in the assessed outcomes, as shown in the first part of this Discussion section. However, the limitations, such as the lack of quantitative analysis and the low quality of existing studies, significantly reduce the strength of our findings. Therefore, despite being systematically conducted, this research alone is not conclusive about the effectiveness of PCs’ applications in the assessed pathologies, thus requiring a high-quality clinical trial to obtain more valid responses to our questions.

## 5. Conclusions

The use of PCs in clinical medicine has expanded into many different medical fields. Many studies covered in this systematic review describe PCs’ potential usefulness in the management of a variety of diseases and conditions. Although PCs can be considered a potentially effective and safe alternative for difficult-to-treat urological disease conditions such as HC and US, the great variability and the not-robust statistical relevance limited their use. So, as repeatedly read in literature [2], in present days, PCs could not yet be strongly recommended in routinary onco-urology clinical practice, mostly especially in those diseases in which effective treatments are already available (for example, urethroplasty with oral mucosa graft in US).

That said, what seems clear is that these treatments seem effective in the small number of cases shown here, and they do not show any AE regardless of the treatment outcomes, which sets a precedent for safely conducting wider clinical trials, as previously intended, and even for the regenerative applications of PCs in former oncological patients. Therefore, for now, we limit our assessment by saying that, for patients who have failed previous treatments for their condition, PCs in general, and particularly PL or liquid PRP for the various cystitis and PRF for US, may play a larger role in disease management. In fact, even today, we can say that PC therapy provides an additional therapeutic option for patients who might otherwise face classical, radical treatments like cystectomy for HC or the placement of a suprapubic catheter for recurrent US. These radical interventions can significantly worsen the patient’s QoL, making minimally invasive options like, for example, bladder instillations of liquid PRP highly valuable.

For these reasons, it sounds reasonable to assume that future high-quality clinical trials are mandatory in this field because achieving the needed safety for such promising and cost-effective treatment might be of paramount importance for onco-urology patients.

## Figures and Tables

**Table 2:** Results of the systematic review of the literature.

1st Author (Year)	ID [cit.]	Topic (Type)	Nr. of Study Subjects	PC	Follow-Up	Treatment Course	QA
Abbruzzese L., et al. (2023)	PMID: 37263885 [20]	*AC* (clinical study = human case series)	9 humans	*PL*	3 months during treatment	**1st month**: 3 treatments × week **2nd month**: 2 treatments × week **3rd month**: 1 treatment × week	Grade C, level 4—6 points out of 9
Chen Y.-H., et al. (2020)	PMID: 32521683 [29]	*CYP-induced cystitis* (pre-clinical study)	30 female rats, randomized in 5 groups of 6 subjects: saline control CYP treated with saline CYP treated with HA CYP treated with PRP CYP treated with PRP and HA	PRP	3 days after treatment	**Day 0**: saline or CYP application **Day 1**: treatments **Day 4**: rats sacrifice	11 points out of 20
Dönmez M.İ., et al. (2016)	PMID: 27706013 [16]	*IC + HC* (pre-clinical study)	36 male rabbits, randomized in 6 groups of 6 subjects: Saline Saline and PRP HCl HCl and PRP CYP CYP and PRP	PRP	2 days after treatment	**Day 0**: saline, HCl, or CYP application **Day 1**: treatment of group 6 **Day 2**: treatment of groups 2 + 4 **Day 3**: group 5 + 6 sacrifice **Day 4**: other groups sacrifice	14 points out of 20
Jhang J.F., et al. (2017)	PMID: 29265766 [7]	*IC + others (clinical study)*	*///*	*///*	*///*	*///*	*Grade D, level 5*
Ke Q.S., et al. (2019)	PMID: 31258287 [1]	*Regenerative urology* (review)	///	///	///	///	Grade D, level 5
Kuo H.-C., et al. (2023)	PMID: 37702275 [12]	*IC + others* (review)	///	///	///	///	Grade D, level 5
Masieri L., et al. (2019)	PMID: 31321678 [30]	*HC* (clinical study = human case series)	10 humans	Platelet gel	1 month after treatment	**Day 0**: treatment **1 month**: follow-up	Grade C, level 4—4 points out of 9
Ninan N., et al. (2014)	PMID: 25500273 [31]	*Regenerative urology* (review)	///	///	///	///	Grade D, level 5
Ozyuvali E., et al. (2016)	PMID: 27917804 [32]	*CYP-induced HC* (pre-clinical study)	24 female rats, randomized in 4 groups of 6 subjects: Control Sham (saline) CYP CYP + PRP	PRP	6 h after treatment	**Day 0**: saline or CYP application + group 1 sacrifice **Day 1**: treatment of group 4 + groups 2 and 3 sacrifice **Day 1, 6 h after treatment**: group 4 sacrifice	11 points out of 20
Saade A., et al. (2020)	PMID: 32526327 [19]	*HC* (review)	///	///	///	///	Grade D, level 5
Soyer T., et al. (2013)	PMID: 24314201 [8]	*Urethral regeneration* (pre-clinical study)	18 rats, divided into 3 groups of 6 subjects: Control Sham (*Vicryl*) PRF	PRF	1 day after treatment	**Day 0**: sampling and sacrifice of group 1 + treatment of groups 2 and 3 **Day 1**: sampling and sacrifice of groups 2 and 3	8 points out of 20
Spartalis E., et al. (2018)	PMID: 29984344 [33]	*PRP in onco-urology* (commentary)	///	///	///	///	Grade D, level 5
Trama F., et al. (2021)	PMID: 34680774 [28]	*IC + others* (review)	///	///	///	///	Grade D, level 5
Yáñez-Castillo Y.M., et al. (2022)	DOI: 10.56434/j.arch.esp. urol.20227508.98 [2]	*PRP in urology* (review)	///	///	///	///	Grade D, level 5

## Data Availability

Data are contained within the article (see Section 2.4 Data Extraction and Interpretation) and Supplementary Materials.

## Supplementary Materials

The following supporting information can be downloaded at https://www.mdpi.com/article/10.3390/ijms251910683/s1.
