# Supplementary figures and images for: Applications of Platelet Concentrates (PCs) in Regenerative Onco-Urology: A Systematic Review of Literature
Source: Int J Mol Sci. 2024 Oct 4;25(19):10683. doi: 10.3390/ijms251910683 (PMC11477022; doi:10.3390/ijms251910683)

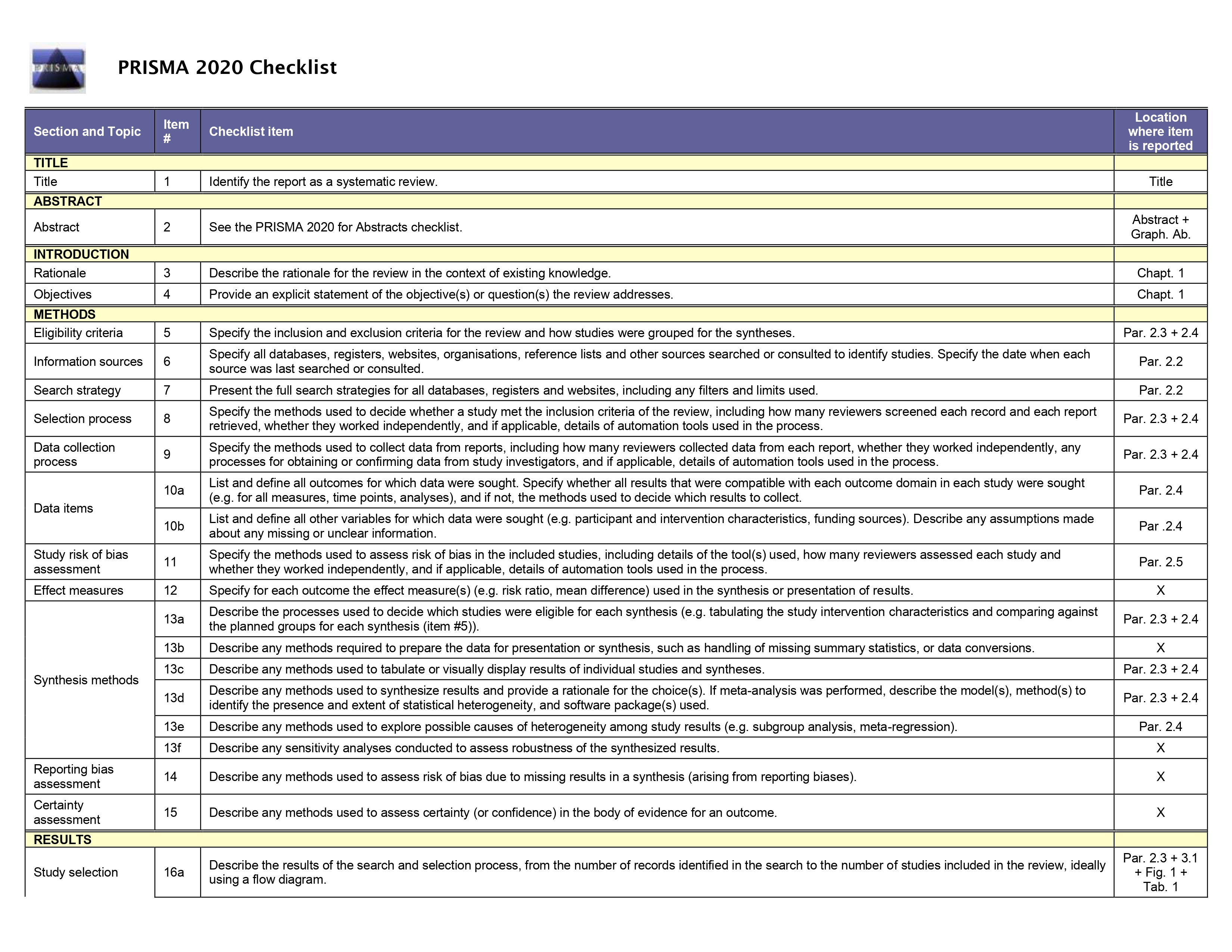

Supplement: Supplementary file 1 [file ijms-25-10683-s001.zip › 05 - New Supplementary Figure 1.tif]

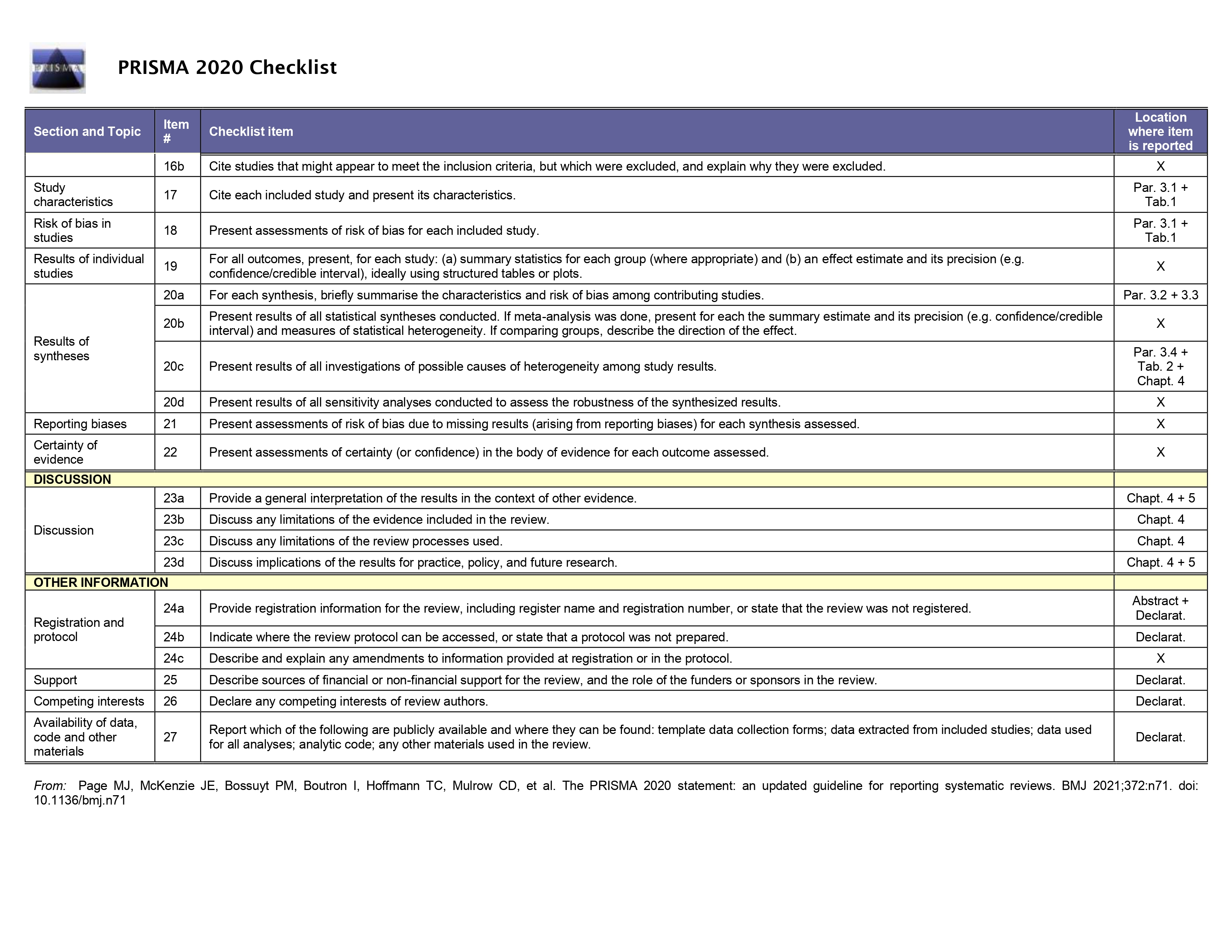

Supplement: Supplementary file 1 [file ijms-25-10683-s001.zip › 05 - New Supplementary Figure 2.tif]
